# Could we reduce renal toxicity of [^177^Lu]Lu-PSMA radioligand therapy with furosemide?

**DOI:** 10.1007/s00259-026-07865-8

**Published:** 2026-04-01

**Authors:** Sabin G. Pop, Klaus Strobel, Jakob Heimer, Chiara Baumann, Aurelius Omlin, Tatjana Leike, Noel Spielhofer, Cristina E. Popescu, Andrea Zander, Irene A. Burger

**Affiliations:** 1 Department of Nuclear Medicine, Kantonsspital Baden, Affiliated Hospital for Research and Teaching of the Faculty of Medicine of the University of Zurich, Baden, 5404 Switzerland; 2 Department of Nuclear Medicine and Radiology, Luzerner Kantonsspital, University Teaching and Research Hospital, University of Lucerne, Lucerne, Switzerland; 3 Digital Trial Intervention Plattform (dTIP), ETH Zurich, Zurich, Switzerland; 4 Onkozentrum Zurich, University of Zurich and Tumorzentrum Hirslanden Zurich, Zurich, Switzerland; 5 Department of Nuclear Medicine, University Hospital Zurich, University of Zurich, Zurich, Switzerland; 6 Department of Health Sciences and Technology, ETH Zurich, Zurich, Switzerland

**Keywords:** Radioligand therapy, mCRPC, Diuretics, Nephrotoxicity, Quantitative SPECT

## Abstract

**Purpose:**

Impaired renal function after [^177^Lu]Lu-PSMA radioligand therapy (RLT) is a growing concern as the field moves toward earlier applications. Little is known how renal function could be protected. This study evaluates the impact of furosemide administered shortly after [^177^Lu]Lu-PSMA RLT, based on long-term follow-up of renal function and quantitative assessment of renal uptake.

**Methods:**

Retrospective analysis of patients treated with a least 4 cycles of [^177^Lu]Lu-PSMA at two Swiss RLT centers with 40 mg furosemide 30 min after injection (CenA) and without (CenB). CenA used [^177^Lu]Lu-PSMA-I&T and [^177^Lu]Lu-PSMA-617, while CenB used only [^177^Lu]Lu-PSMA-I&T. A mixed-effects model was used to evaluate eGFR and PSA change per cycle in the first cohort. To increase the number for renal uptake of [^177^Lu]Lu-PSMA-I&T in CenA, supplementary patients with less than 4 cycles were included for quantitative SPECT assessment.

**Results:**

In CenA, 24 patients with 5.4±0.8 doses (17 [^177^Lu]Lu-PSMA-617 / 7 [^177^Lu]Lu-PSMA-I&T) were followed over 202.0±58 days. In center CenB 20 patients with 5.4±0.9 doses were followed over 210.6±48 days. CenB showed a significant drop in eGFR per cycle of -2 ml/min/1.73m^2^ (CI − 2.8/−1.1), *p* < 0.001, while CenA showed no significant decrease per cycle, with 0.3 ml/min/1.73m^2^ (CI − 0.6/1.1), *p* = 0.587. For quantitative analysis 20 patients with [^177^Lu]Lu-PSMA-I&T in CenB had a mean SUV_peak_ of 7.7±3.1, while 16 patients in CenA with [^177^Lu]Lu-PSMA-I&T had a mean SUV_peak_ of 7.1±3.0 and 17 patients with [^177^Lu]Lu-PSMA-617 had a mean of 6.2±1.8.

**Conclusion:**

Furosemide may reduce tracer accumulation in the kidneys after [^177^Lu]Lu-PSMA RLT. This might explain the mostly stable eGFR values observed in CenA, while there was a significant drop in CenB, without furosemide. If diuretics could reduce renal toxicity without impairing efficacy warrants further prospective evaluation.

**Supplementary Information:**

The online version contains supplementary material available at https://doi.org/10.1007/s00259-026-07865-8.

## Introduction

The increased expression of Prostate-specific-membrane Antigen (PSMA) on prostate cancer (PCa) cells has become a target for various diagnostic and therapeutic approaches. One being Lutetium-177 radioligand therapy (RLT), which is becoming increasingly important as a therapy option in patients with metastatic castration-resistant prostate cancer (mCRPC). PSMA-targeting RLT offers well-tolerated, targeted therapy with limited side effects [1]. Therefore, applications and indications are rapidly expanding [2, 3].

Following the phase 3 VISION and PSMAfore trial [^177^Lu]Lu-PSMA RLT has been approved for mCRPC before or after chemotherapy, where it has shown both enhanced progression-free survival in both trials and overall survival only in the VISION trial in patients with mCPRC [1, 3]. Consequently, the question becomes apparent, whether an earlier indication of PSMA-RLT might be beneficial. Early studies suggested no significant decrease in renal function using [^177^Lu]Lu-PSMA-I&T even when measured via functional renal scintigraphy in addition to serum biomarkers [4]. However, the follow-up in this cohort was limited to the duration of the RLT, with only 17 patients receiving 5 or more cycles [4]. Furthermore, initial safety studies for using 6.8 GBq [^177^Lu]Lu-PSMA-I&T did not indicate any relevant renal impairment, even in patients with higher cumulative doses than 23 Gy [5]. The dosimetry study from the VISION trial revealed a mean absorbed dose in the kidneys was 15 ± 5.8 Gy, concluding that a total of 44.4 GBq on 6 cycles should not lead to renal toxicity, within 5.52 months [6]. Similarly, a retrospective study of 110 patients, revealed no correlation between cumulative renal dose and decrease in glomerular filtration rate (GFR) during a follow up period of 6 weeks after RLT, they did reduce the dose to 5.5–6.5 GBq per cycle for patients with solitary kidneys or pre-existing nephropathy (eGFR < 60 ml/min/1.73m^2^) [7].

Recently the data of a randomized phase 3 trial in metastatic hormone sensitive prostate cancer was presented at ESMO 2025, showing a progression free survival benefit for patients treated not only with androgen deprivation therapy and an androgen receptor pathway inhibitor but additional 6 cycles of 7.4 GBq [^177^Lu]Lu-PSMA-617 (PSMAddition Scott T. et al., ESMO 2025). However, the data also showed that side effects and a decrease in well-being were observed in the therapy arm beyond the expected dry mouth, which affected 46.5% of patients. Grade 1–2 cytopenia was twice as common in the therapy arm, and renal events occurred in 7.1% of patients compared to 4.6% in the control arm.

Therefore, especially for earlier applications, long term side effects such as decrease of renal function might become more relevant. Close attention should therefore be paid to renal damage as one of the more identified side effects of PSMA targeting RLT. Furthermore, when considering combination therapies involving either chemotherapy or hormone therapy, it is important to pay attention to the potential loss of kidney function.

It has been suggested that the type of radio ligand used, [^177^Lu]Lu-PSMA-617 or [^177^Lu]Lu-PSMA-I&T, might influence the extent of renal functional impairment. This assumption is based on dosimetry data showing a slightly higher renal uptake for [^177^Lu]Lu-PSMA-I&T with a mean absorbed kidney dose of 0.73 ± 0.33 Gy/GBq [5] compared to 0.44 ± 0.21 Gy/GBq [6] with [^177^Lu]Lu-PSMA-617. Both ligands are small-molecule inhibitors that target the enzymatic pocket of PSMA. However, their molecular structures are dissimilar, with variance in the overall hydrophilicity and protein binding, leading to divergent pharmacokinetic and dosimetry profiles.

Nevertheless, reports comparing renal toxicity between both RLT ligands could not document a significant difference in decrease in GFR [8, 9]. However, increasing long term follow-up data show that a moderate decrease in eGFR (30–40%) can be observed in 45% of the patients after one year [10]. Furthermore, first reports on severe radiation nephropathy 12–17 months after therapy in up to 10% of patients receiving > 6 cycles of [^177^Lu]Lu-PSMA-I&T have been published [11].

To reduce renal radiation exposure, strategies to increase clearance from the kidneys might improve tolerability. Furosemide (Lasix©), a loop diuretic, inhibits the reabsorption of electrolytes in the proximal and distal tubules, and increasing the volume of excretion, without increasing the renal perfusion or GFR [12]. The faster excretion of [^68^Ga]Ga-PSMA-11 from the renal system has been well established for imaging, therefore administration of 20 mg furosemide i.v. just prior or after the administration of hydrophilic PSMA PET ligands ([^68^Ga]Ga-PSMA-11, [^18^F]F-DCFPyl) is part of the EANM guidelines for PSMA PET/CT imaging [13]. Therefore, Topal et al. suggested the use of 40 mg furosemide after therapy in patients without contraindications [7]. Nevertheless, the use of furosemide for [^177^Lu]Lu-PSMA RLT was not part of the protocol in the initial trials.

The aim of this two-center retrospective analysis is to evaluate the risk–benefit profile of furosemide administered shortly after [^177^Lu]Lu-PSMA RLT, based on long term follow-up of renal function, but also quantitative assessment of renal uptake based on SPECT/CT scans performed 48 h after each therapy cycle.

## Methods

### Patient cohort

This retrospective, two-center comparative study was designed to evaluate the impact of furosemide administration shortly after [^177^Lu]Lu-PSMA RLT on renal function and therapeutic efficiency. Data were collected from two Swiss nuclear medicine institutes: Kantonsspital Baden (CenA) and Luzerner Kantonsspital (CenB), with approval from the local ethics committee (BASEC 2025 − 00461). Inclusion criteria were a signed general consent, a minimum of four cycles of [^177^Lu]Lu-PSMA RLT according to the inclusion criteria of the VISION trial (mCRPC with one/two lines of taxane or unfit for chemotherapy) and no prior renal obstruction or nephrectomy. Renal function was assessed using the Chronic Kidney Disease Epidemiology Collaboration (CKD-EPI) formula to estimate the GFR (eGFR) before each treatment cycle and after therapy. The formula is as follows: eGFR = 141×min(Scr/k,1)^a^ × max(Scr/k,1)-1.209 × 0.993^age^ (with k = 0.9, a = -0.411, where min and max represent the minimum respectively maximum values of Scr/k or 1) [14]. A decrease of at least 30% in eGFR was defined as clinically significant [15, 16].

Except for the administration of 40 mg furosemide, 30 min after therapy application at CenA, the therapeutic protocols were identical. The CenA cohort included patients treated with either [^177^Lu]Lu-PSMA-I&T or [^177^Lu]Lu-PSMA-617. The CenB cohort exclusively comprised patients treated with [^177^Lu]Lu-PSMA-I&T. Given this imbalance in the two radioligands used, and the significantly lower number of patients undergoing [^177^Lu]Lu-PSMA-I&T with furosemide, for the quantitative assessment of renal and hepatic uptake a supplementary cohort of CenA patients who had undergone fewer than four therapy cycles was included in the secondary analysis to achieve a balanced distribution of patients.

### Therapy protocol

Patients received [^177^Lu]Lu-PSMA therapy, administered at 7.4 GBq per cycle with 6-week intervals (± 1 weeks). Patients with a solitary kidney, were excluded from this analysis. Extended (> 4) cycles were continued if both biochemical and imaging responses were observed and no clinical or biochemical deterioration, due to potential side effects occurred. The decision to proceed with extended therapy was made by a multidisciplinary board.

Biochemical response was assessed by a PSA decline of at least 50% 8–12 weeks after starting treatment. Imaging based response was assessed using quantitative [^177^Lu]Lu-PSMA SPECT/CT after each cycle. [^177^Lu]Lu-PSMA therapy was administered as a slow intravenous injection (3–5 min) according to the European Association of Nuclear Medicine (EANM) guidelines [17]. With each treatment, patients without history of cardiac conditions received intravenous hydration with 500–1000 ml of 0.9% NaCl. Sufficient hydration was recommended in the weeks following treatment.

The therapy protocol was identical, except for the administration of 40 mg furosemide 30 min after RTL injection in CenA, which was not performed in CenB. CenA used [^177^Lu]Lu-PSMA-I&T and [^177^Lu]Lu-PSMA-617, while CenB only used [^177^Lu]Lu-PSMA-I&T.

### Outcome analysis

We screened the hospital information system for creatinine and eGFR values, as well as PSA follow-up, during therapy and follow-up. For external patients, referring physicians were contacted to obtain latest renal function values.

### Imaging protocol

In both centers, the post-therapy scan was performed on the second day (48 h) after RLT application. Both centers used a Symbia Intevo Bold SPECT/CT (Siemens Healthineers, Erlangen, Germany) with a MELP collimator. Utilizing the low-energy gamma emitted by Lu-177 (Eγ max of 208 keV)., The examination consisted of whole-body planar scintigraphy with ventral and dorsal acquisitions, followed by partial-body SPECT/CT from the parotid gland level to the patient’s pelvis (2–3 bed positions), with a SPECT slice thickness of 2.0 mm, a matrix of 256 × 256 and an acquisition time of approximately 15 min per bed (10 s. per View). Image reconstruction was performed using xSPECT_Quant_ for quantitative assessment of tracer distribution. The reconstruction parameters were identical for both centers, eight iterations with four subsets and 3D Gaussian full width at half maximum (FWHM) filtering (8.0 mm).

### Image analysis

**Fig. 1:**
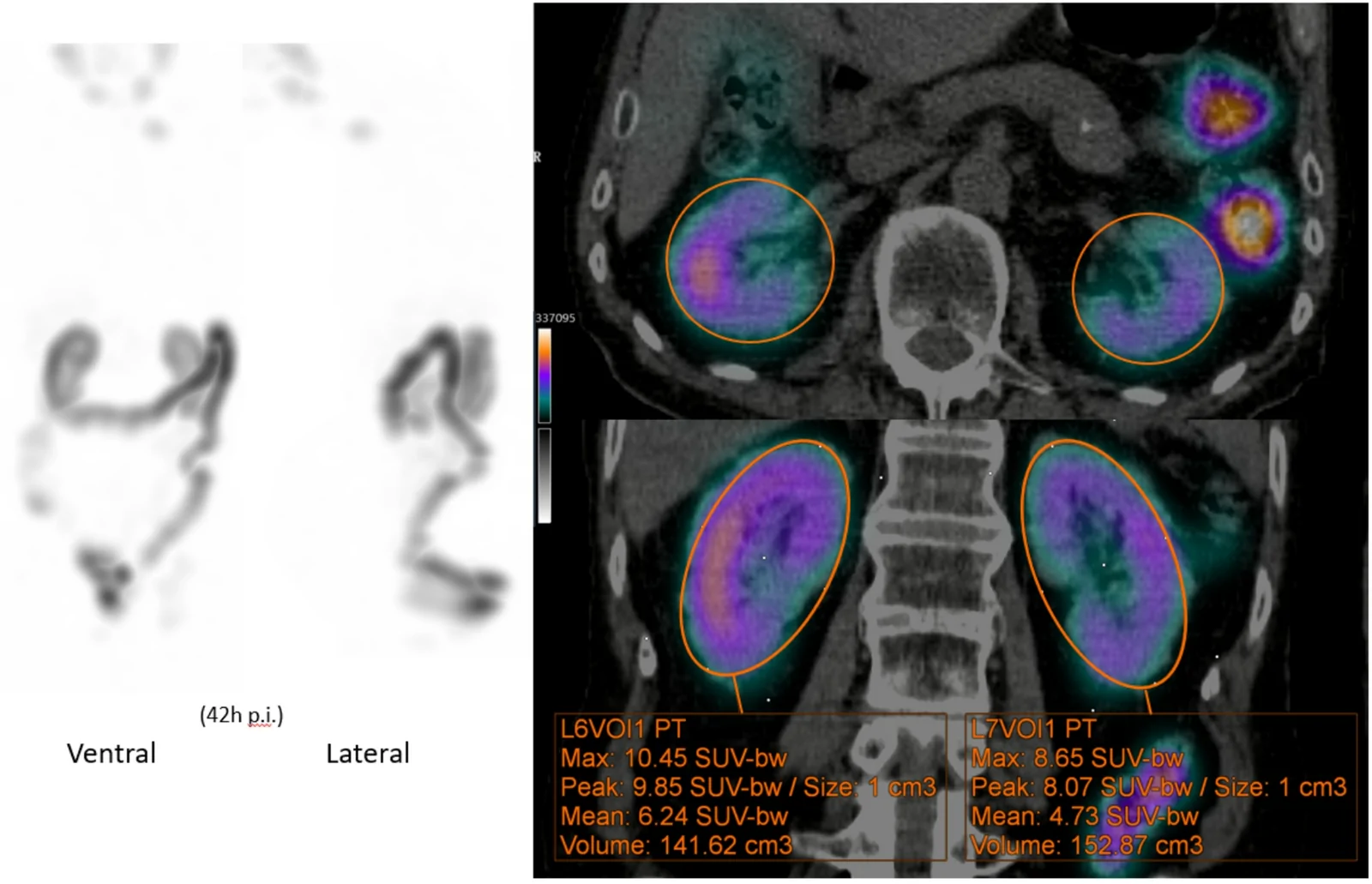
Illustration of SPECT image analysis using spheroid volumes of interest covering the entire kidneys, without inclusion of extra-renal tracer accumulation. For further analysis SUVpeak was selected

In a central readout all scans were reviewed for diagnostic quality. If scan quality was approved, volumes of interest (VOI) were placed around the kidneys by a nuclear medicine resident or medical student (SGP, CB) using Syngo.Via VB80D©, making sure that there is no pathological uptake of adjacent metastasis within the VOI (Fig. 1). All VOIs and measurements were double checked by a senior nuclear medicine physician and radiologist with over 16 years of experience (IAB). To quantify [^177^Lu]Lu-PSMA accumulation SUV_peak_ was selected to minimize impact of VOI size on SUV_mean_ and reduce variability over SUV_max_ [18, 19].

### Statistics

Continuous variables were summarized as mean ± standard deviation (SD) and categorical variables as frequencies (%). Differences in baseline values were assessed using the Wilcoxon rank-sum test or Fisher’s exact test. Longitudinal changes in eGFR and log_2_-transformed PSA were analyzed using linear mixed-effects models (LMM). These models included fixed effects for Therapy cycle, Center/Substance Group, dose, as well as age and baseline values. Interactions between cycle and Substance, baseline values, and dose were modeled. A random intercept was included for patient_id to account for repeated measures. Pairwise contrasts of the slopes were calculated using estimated marginal means. Differences in organ uptake (SUV_peak_) were analyzed using LMMs adjusted for baseline eGFR, cycle, and age. All analyses were performed using R (version 4.4.1). P-values < 0.05 were considered statistically significant.

## Results

### Patient cohort

In the first cohort, including only patients receiving at least four cycles of [^177^Lu]Lu-PSMA RLT, 24 patients were identified in CenA with a mean of 5.4±0.8 doses (17 [^177^Lu]Lu-PSMA-617 and 7 [^177^Lu]Lu-PSMA-I&T), average follow-up was 202.0±58 days. In CenB 20 patients with a mean of 5.4±0.9 doses were identified and followed over a mean of 210.6±48 days. Baseline eGFR and PSA values were not significantly different between both centers. The mean administered dose was 7.3 ± 0.1 GBq (CenA) and **7**.4 ± 0.2 GBq (CenB), this difference reached statistical significance despite the similar values (*p* = 0.002) (Table 1).

**Table 1:** Patient characteristics for outcome analysis

Characteristic	CenA,^1^ *N* = 24	CenB,^1^ *N* = 20	*p*-value^2^
Age	76.6 (7.2)	73.9 (8.1)	0.2
Baseline PSA	124.3 (181.8)	138.7 (220.5)	0.6
Baseline eGFR	71.0 (15.4)	74.4 (16.0)	0.5
Total Cycles			0.7
4	5 (21%)	5 (25%)	
5	5 (21%)	2 (10%)	
6	14 (58%)	13 (65%)	
After therapy Follow-up (Days)	228.8 (215.8)	301.3 (234.2)	0.3
Baseline Kidney SUV_peak_	6.4 (1.8)	7.7 (3.1)*	0.2
Baseline Liver SUV_peak_	0.9 (0.4)	0.8 (0.6)*	0.2
Dose (GBq)	7.3 (0.1)	7.4 (0.2)	0.002

^1^Mean (SD); n (%)

^2^Wilcoxon rank sum test; Fisher’s exact test; Wilcoxon rank sum exact test

*Not available in 2 patients

### Impact on renal function and PSA

**Fig. 2:**
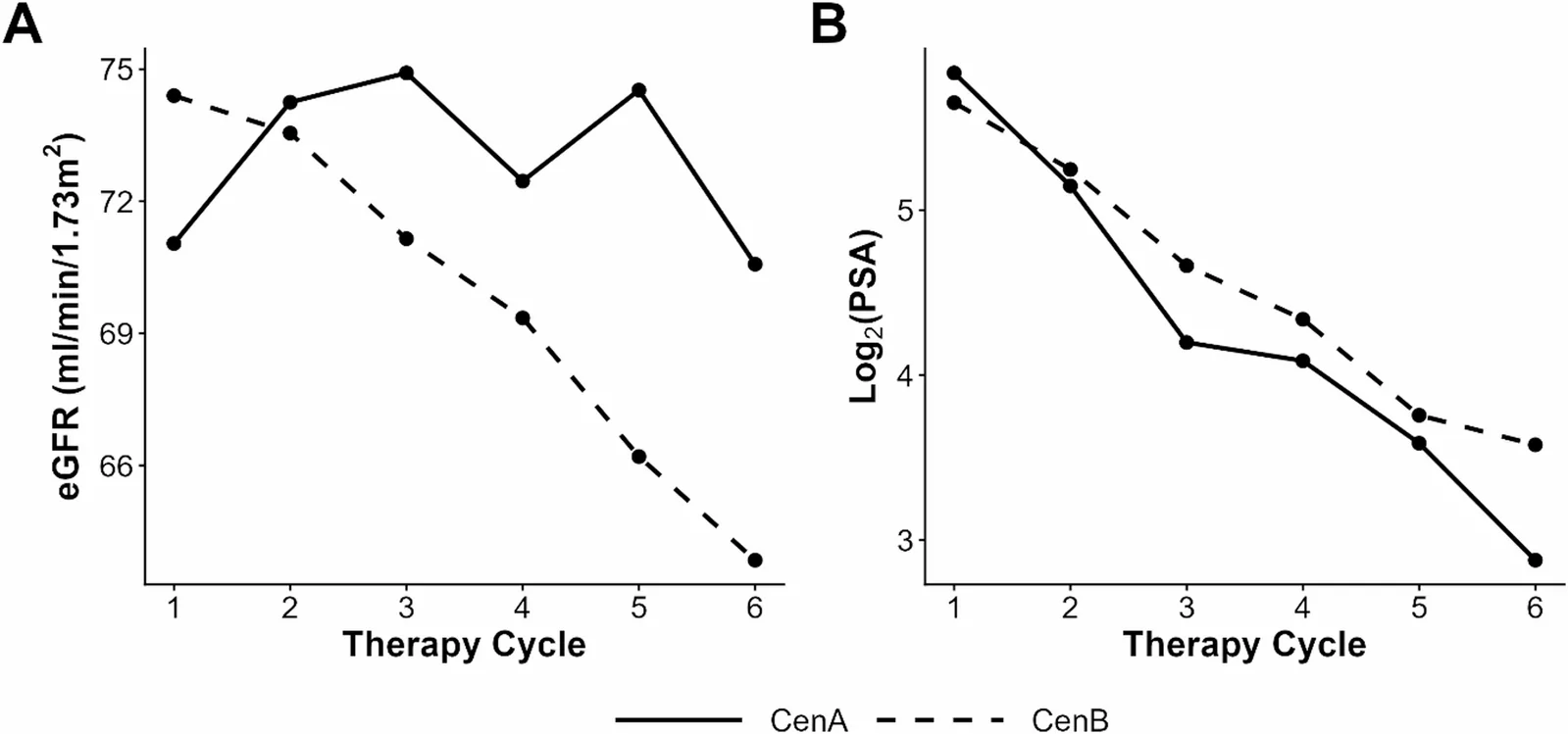
Trajectories for eGFR (**A**) and log2-transformed PSA (**B**) using linear mixed-effects models. These models included fixed effects for therapy cycle, Center/Substance Group, and their interactions, as well as age and baseline values (eGFR, PSA)

Based on the mixed-effects model, there was no significant decrease in eGFR per cycle in CenA with the addition of furosemide, with 0.3 ml/min/1.73m^2^ (CI − 0.6, 1.1), *p* = 0.587, while a significant drop in eGFR per cycle was observed in CenB of -2.0 ml/min/1.73m^2^ (CI − 2.9, − 1.0), *p* < 0.001 *(*Fig. 2A; Table 2). The average eGFR decrease between baseline and last follow up in CenA was − 6.5±19 ml/min/1.73m^2^, while in CenB an average decrease of – 20±20 ml/min/1.73m^2^ was observed. Neither dose nor age had an impact on eGFR changes. In contrast, PSA decline was observed in both centers, with a more pronounced decline in CenA compared to CenB (log(PSA) of CenA − 0.56 (CI − 0.68, − 0.43) and CenB − 0.36 (CI-0.51, − 0.21), (*p* = 0.047)) (Table 2; Fig. 2B).

**Table 2:** Outcome trends eGFR and PSA

Metric	Group	Slope [95% CI]	*p*-value (vs. 0)	*p*-value (vs. Ref)
eGFR Slope	CenA	0.3 [-0.6, 1.1]	0.587	—
	CenB	-2.0 [-2.9, -1.0]	< 0.001	< 0.001
log2(PSA) Slope	CenA	-0.56 [-0.68, -0.43]	< 0.001	—
	CenB	-0.36 [-0.51, -0.21]	< 0.001	0.047

### Impact on renal and hepatic uptake

To consider potential differences between both RLT ligands in distribution for the quantitative analysis of kidney uptake, additional patients with less than 4 cycles were included in CenA, to create a more balanced cohort. This resulted in 16 patients in CenA with [^177^Lu]Lu-PSMA-I&T and 17 receiving [^177^Lu]Lu-PSMA-617 compared to 20 patients in CenB treated with [^177^Lu]Lu-PSMA-I&T without furosemide (Table 3). The mean renal uptake 48 h after injection of [^177^Lu]Lu-PSMA-I&T at baseline with furosemide was SUV_peak_ 7.1±3.0, while in CenB the uptake of [^177^Lu]Lu-PSMA-I&T without furosemide was higher 7.7±3.1. Also, for [^177^Lu]Lu-PSMA-617 with furosemide the uptake was lower compared to CenB with SUV_peak_ 6.2±1.8.

**Table 3:** Patient characteristics for quantitative analysis

Characteristic	CenA PSMA-617,^1^ *N* = 17	CenA PSMA-I&T,^1^ *N* = 16	CenB PSMA-I&T,^1^ *N* = 20	*p*-value^2^
Age	76.4 (7.7)	78.6 (6.5)	73.9 (8.1)	0.15
Baseline PSA	92.1 (116.7)	244.7 (372.6)	138.7 (220.5)	0.5
Baseline eGFR	71.3 (17.4)	72.7 (12.3)	74.4 (16.0)	0.8
Total Cycles				0.003
1	0 (0%)	4 (25%)	0 (0%)	
2	0 (0%)	3 (19%)	0 (0%)	
3	0 (0%)	2 (13%)	0 (0%)	
4	3 (18%)	2 (13%)	5 (25%)	
5	3 (18%)	2 (13%)	2 (10%)	
6	11 (65%)	3 (19%)	13 (65%)	
Total Follow-up (Days)	452.5 (264.6)	217.9 (193.0)	511.9 (249.3)	0.001
Therapy Duration (Days)	211.1 (61.5)	90.1 (90.2)	210.6 (48.3)	< 0.001
After therapy Follow-up (Days)	241.4 (240.2)	127.8 (125.6)	301.3 (234.2)	0.062
Baseline Kidney SUV_peak_	6.2 (1.8)	7.1 (3.0)	7.7 (3.1)*	0.3
Baseline Liver SUV_peak_	1.0 (0.4)	0.6 (0.3)	0.8 (0.6)*	0.016

^1^Mean (SD); n (%)

^2^Kruskal-Wallis rank sum test; Fisher’s exact test

*Not available in 2 patients

**Fig. 3:**
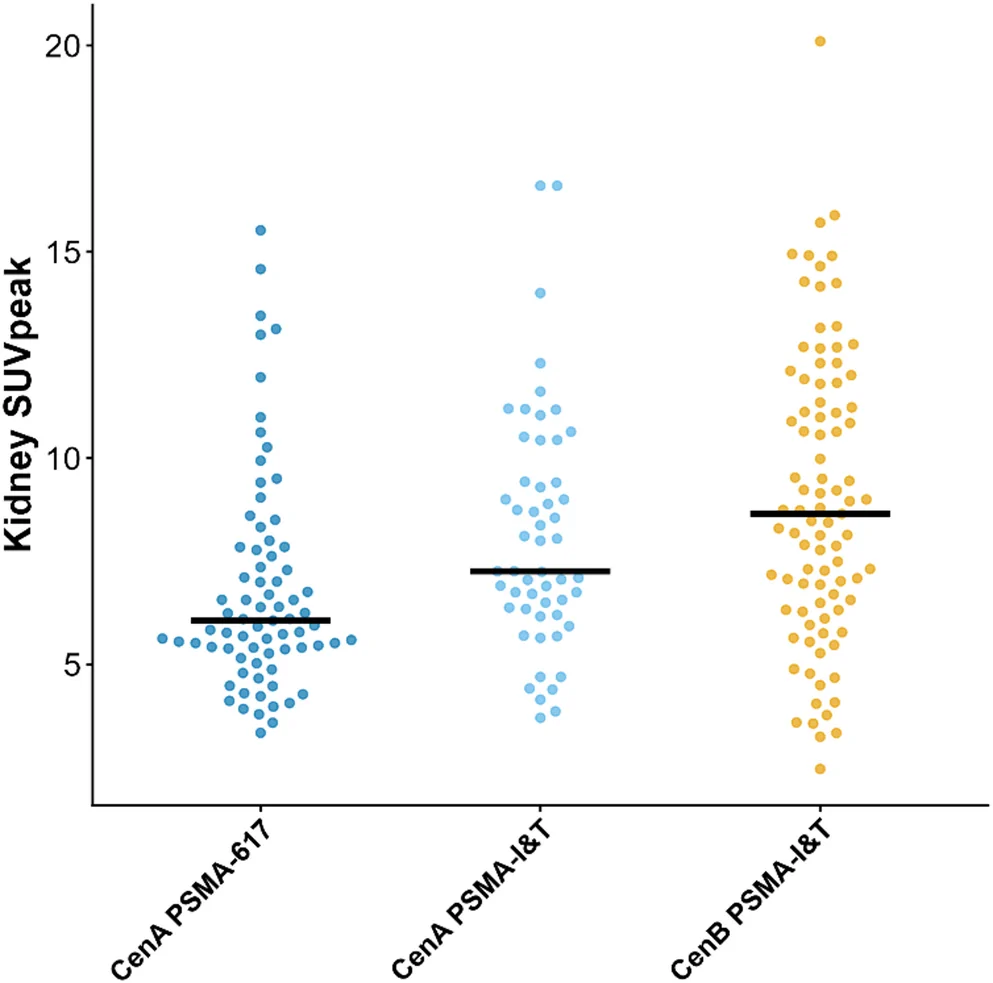
Renal uptake of [^177^Lu]Lu-PSMA-617 with furosemide was lower compared to [^177^Lu]Lu-PSMA-I&T with furosemide in CenA (not reaching significance). Comparing [^177^Lu]Lu-PSMA-I&T between both center, furosemide reduced the uptake, also not reaching statistical significance

Pairwise comparison of the mixed-effects model for renal uptake, reached significance only between [^177^Lu]Lu-PSMA-617 in CenA with furosemide, in comparison to [^177^Lu]Lu-PSMA-I&T in CenB without furosemide (estimated difference of 1.69 [0.20,33.18], *p* = 0.027). There was no significant difference between [^177^Lu]Lu-PSMA-617 and [^177^Lu]Lu-PSMA-I&T if given diuretics, or for [^177^Lu]Lu-PSMA-I&T with or without diuretics *(*Fig. 3; Table 4).

**Table 4:** Kidney SUVpeak analysis

Term	Estimate_CI	*p*-value
(CenA PSMA-617) - (CenA PSMA-I&T)	-0.52 [-1.86, 0.82]	0.450
(CenA PSMA-617) - (CenB PSMA-I&T)	-1.69 [-3.14, -0.23]	0.027
(CenA PSMA-I&T) - (CenB PSMA-I&T)	-1.17 [-2.67, 0.33]	0.133

For liver accumulation there was no significant difference for [^177^Lu]Lu-PSMA-I&T in SUV_peak_ between both centers with 0.6±0.3 in CenA, compared to 0.8±0.6 for CenB.

### Significant eGFR decrease

A significant decrease of more than 30% in eGFR was observed in 2/24 (8%) patients in CenA. In both cases, eGFR was not impaired at the beginning of the therapy with a creatinine of 103 µmol/l / eGFR 63 ml/min/1.73m^2^ (in one patient) and a creatinine of 80 µmol/l and eGFR of 87 ml/min/1.73m^2^ (in the other). Four patients were treated with an initial eGFR < 60 ml/min/1.73m^2^ (range 46–56) without dose reduction. Over a mean follow-up time of 301±120 days none of these patients had a relevant decline in renal function.

In CenB a decline of more than 30% in eGFR was observed in 5/20 (25%). Only one of the five patients had an eGFR < 60 ml/min/1.73m^2^ at the first cycle (53 ml/min/1.73m^2^), the other 4 patients had creatinine values in normal range (66–91 µmol/l). The patient in CenB with the lowest renal function at first cycle with a creatinine of 151 and an eGFR of 36 ml/min/1.73m^2^ remained stable over 4 cycles and a follow time of 167 days.

## Discussion

Increasing urinary volume with diuretics given shortly after injection seems to protect renal function during PSMA-RLT. This is suggested by the significant difference in eGFR reduction on long-term follow-up, seen between two centers that followed the same RLT protocol except the additional administration of 40 mg furosemide 30 min after injection at CenA. This is also reflected with a trend to higher uptake in the kidneys 48 h after injection on post-therapy scans in patients treated with [^177^Lu]Lu-PSMA-I&T without furosemide, compared to [^177^Lu]Lu-PSMA-I&T with diuretics. This is the first analysis looking into a potential impact of diuretics on renal function during [^177^Lu]Lu-PSMA-directed RLT with a long term follow up.

Our quantitative analysis revealed a stepwise increase in accumulation from [^177^Lu]Lu-PSMA-617 to [^177^Lu]Lu-PSMA-I&T with furosemide and to [^177^Lu]Lu-PSMA-I&T without furosemide. Both steps did not reach significance but are in line with the hypothesis that furosemide is reducing the activity for [^177^Lu]Lu-PSMA-I&T and that [^177^Lu]Lu-PSMA-I&T has a higher renal accumulation compared to [^177^Lu]Lu-PSMA-I&T-617. This finding is consistent with the higher tracer accumulation in post-therapy dosimetry for [^177^Lu]Lu-PSMA-I&T compared to [^177^Lu]Lu-PSMA-617 observed by other groups [8, 20]. Schuchardt et al. compared dose distribution and toxicity in 138 patients treated without diuretics. 51 received [^177^Lu]Lu-PSMA-I&T and 87 had [^177^Lu]Lu-PSMA-617, with the result that [^177^Lu]Lu-PSMA-I&T had a marginally higher dose of 0.8 Gy/GBq compared to [^177^Lu]Lu-PSMA-617 with 0.9 Gy/GBq. However, this did not result in a significant difference in renal toxicity between both groups during the observation period [8]. Similarly Hartrampf et al. did not observe a significant difference in renal toxicity in a bicentric matched-pair analysis between 55 patients treated with [^177^Lu]Lu-PSMA-617 and 55 undergoing RLT with [^177^Lu]Lu-PSMA-I&T with comparable overall survival [9].

In our cohort, a significant decrease of more than 30% in eGFR was observed in only 8% of patients in CenA, compared to 25% in CenB, despite a higher number of patients with an eGFR < 60 ml/min/1.73m^2^ at first cycle in CenA (*n* = 4) compared to CenB (*n* = 1). This is consistent with previous observations, that there is only a weak association between a decline of more than 30% after [^177^Lu]Lu-PSMA-I&T and baseline eGFR value [21]. Steinfelder et al. found that the mean eGFR was not significantly lower for patients with renal decline, with 70.8±14.7 ml/min/1.73m^2^ compared to 78.3±18.0 ml/min/1.73m^2^ in patients without (*p* = 0.01) [21, 22].

There are chemical differences between [^177^Lu]Lu-PSMA-I&T and [^177^Lu]Lu-PSMA-617. [^177^Lu]Lu-PSMA-I&T was developed for dual labeling with an additional Iodine that could be used for imaging or therapy. The radionuclide conjugation is based on four COO- groups, leading to an overall negative charge of the chelator, this is compensated with a long connecting arm to the binding moiety, rendering [^177^Lu]Lu-PSMA-I&T a bit more polar and less lipophilic compared to [^177^Lu]Lu-PSMA-617 [23, 24]. This might also explain the slightly higher tracer uptake in the liver parenchyma observed for [^177^Lu]Lu-PSMA-617 in our cohort.

Regarding PSA decline, we did not observe any impairment due to the use of furosemide. In the analysis PSA reduction was deeper in CenA compared to CenB. Whether this is due to the higher number of patients undergoing [^177^Lu]Lu-PSMA-617 compared to [^177^Lu]Lu-PSMA-I&T is unclear and would be contradicted with other matched pair analyses that did not observe any differences in outcomes [9].

Potential applications for renal protection during PSMA-directed RLT are sparse. Kristiansson et al. suggest using antioxidant α1-Microglobulin to improve renal function after [^177^Lu]Lu-PSMA-617, but this was tested only in a preclinical model in tumor-bearing mice [25, 26]. Others suggested using Mannitol injections to increase diuresis in a prospective study of 9 patients with only a slight, nonsignificant reduction in renal dose [27]. Given that furosemide is a well-established therapy with limited toxicity and drug interactions, this seems a more practical alternative.

This retrospective, multicenter study had a relatively small size, two different ligands and without multiple timepoint dosimetry. Furthermore, the ligands were not distributed homogeneously between both centers, leading to a cofounder for the analysis. However, Both centers have the same scanner and imaging protocol, using quantitative assessment of [^177^Lu]Lu-PSMA SPECT data. An increasing amount of literature and data suggests that a reasonable approximation for total dose can be achieved, based on single time point quantitative imaging. We therefore considered SUV_peak_ a good surrogate marker to compare renal dose, using centralized readout, identical scanner equipment as well as imaging protocols. Comparative studies on toxicity involving different radioligands are very difficult to interpret. Small cohorts and numerous clinical variables can confound such findings. We therefore chose a linear mixed model to incorporate clinical parameters such as age, basal renal function and number of cycles, to estimate the impact of a single therapy cycle, with or without furosemide, on eGFR.

## Conclusion

Furosemide is reducing tracer accumulation in the kidneys on post-therapy scans of [^177^Lu]Lu-PSMA RLT. Higher renal uptake might explain the significant decrease in eGFR observed in CenB not using furosemide, that was not seen in CenA. Whether diuretics have the potential to reduce renal toxicity without negative impact on antitumor efficacy warrants prospective evaluation.

## Supplementary Information

Supplementary Material 1.

Supplementary Material 2.

Supplementary Material 3.

## Data Availability

The datasets generated during and/or analysed during the current study are available from the corresponding author on reasonable request.
