# Supplementary material for: Could we reduce renal toxicity of [177Lu]Lu-PSMA radioligand therapy with furosemide?
Source: Eur J Nucl Med Mol Imaging. 2026 Apr 1;53(9):5427–35. doi: 10.1007/s00259-026-07865-8 (PMC13652897; doi:10.1007/s00259-026-07865-8)
Supplement: Supplementary file 1 [file 259_2026_7865_MOESM1_ESM.docx]

Appendix: Raw Fixed Effects - Kidney SUV Model

| **effect** | **term** | **estimate** | **std.error** | **statistic** | **df** | **p.value** |
| --- | --- | --- | --- | --- | --- | --- |
| fixed | (Intercept) | 6.82727895 | 4.18757117 | 1.6303673 | 70.35086 | 0.10749 |
| fixed | SubstanceCenA PSMA-I&T | 0.51973701 | 0.67751619 | 0.7671212 | 109.51213 | 0.44466 |
| fixed | SubstanceCenB PSMA-I&T | 1.68725110 | 0.74002900 | 2.2799797 | 46.00738 | 0.02729 |
| fixed | eGFR | -0.05716773 | 0.01523165 | -3.7532203 | 165.60666 | 0.00024 |
| fixed | Cycle | 0.33317785 | 0.08356896 | 3.9868615 | 176.09629 | 0.00010 |
| fixed | Age | 0.04712423 | 0.04725803 | 0.9971686 | 54.26111 | 0.32311 |
