# Supplementary material for: Could we reduce renal toxicity of [177Lu]Lu-PSMA radioligand therapy with furosemide?
Source: Eur J Nucl Med Mol Imaging. 2026 Apr 1;53(9):5427–35. doi: 10.1007/s00259-026-07865-8 (PMC13652897; doi:10.1007/s00259-026-07865-8)
Supplement: Supplementary file 2 [file 259_2026_7865_MOESM2_ESM.docx]

Appendix: Raw Fixed Effects - PSA Model

| **effect** | **term** | **estimate** | **std.error** | **statistic** | **df** | **p.value** |
| --- | --- | --- | --- | --- | --- | --- |
| fixed | (Intercept) | 4.012705060 | 4.5334899483 | 0.88512495 | 183.92076 | 0.37724 |
| fixed | Cycle | -1.447069073 | 0.8825791339 | -1.63959130 | 174.98508 | 0.10289 |
| fixed | Substance_GroupedCenB | -0.419441997 | 0.6068169658 | -0.69121666 | 76.06976 | 0.49153 |
| fixed | Age | 0.019077647 | 0.0340077525 | 0.56097936 | 42.23530 | 0.57778 |
| fixed | baseline_psa | 0.008559366 | 0.0014709044 | 5.81911761 | 66.94297 | 0.00000 |
| fixed | Dose | -0.039991625 | 0.5040046006 | -0.07934774 | 174.24732 | 0.93685 |
| fixed | Cycle:Substance_GroupedCenB | 0.199946737 | 0.1000632555 | 1.99820340 | 172.40906 | 0.04727 |
| fixed | Cycle:baseline_psa | -0.000142512 | 0.0002135635 | -0.66730516 | 170.14020 | 0.50548 |
| fixed | Cycle:Dose | 0.123751031 | 0.1219739075 | 1.01456971 | 175.01371 | 0.31171 |
