# Supplementary material for: Could we reduce renal toxicity of [177Lu]Lu-PSMA radioligand therapy with furosemide?
Source: Eur J Nucl Med Mol Imaging. 2026 Apr 1;53(9):5427–35. doi: 10.1007/s00259-026-07865-8 (PMC13652897; doi:10.1007/s00259-026-07865-8)
Supplement: Supplementary file 3 [file 259_2026_7865_MOESM3_ESM.docx]

Appendix: Raw Fixed Effects - eGFR Model

| **effect** | **term** | **estimate** | **std.error** | **statistic** | **df** | **p.value** |
| --- | --- | --- | --- | --- | --- | --- |
| fixed | (Intercept) | 23.230844113 | 29.52220412 | 0.78689396 | 192.9688 | 0.43231 |
| fixed | Cycle | -1.897767311 | 5.80746620 | -0.32678060 | 184.4190 | 0.74420 |
| fixed | Substance_GroupedCenB | 1.089827031 | 2.95051242 | 0.36936873 | 134.8734 | 0.71243 |
| fixed | Age | -0.205710185 | 0.15917131 | -1.29238231 | 42.4391 | 0.20322 |
| fixed | baseline_eGFR | 0.934384405 | 0.10054913 | 9.29281486 | 104.4206 | 0.00000 |
| fixed | Dose | -0.221794427 | 3.24161211 | -0.06842103 | 182.7803 | 0.94553 |
| fixed | Cycle:Substance_GroupedCenB | -2.222709634 | 0.65130742 | -3.41268897 | 178.1030 | 0.00080 |
| fixed | Cycle:baseline_eGFR | -0.003078498 | 0.02048226 | -0.15030071 | 180.1925 | 0.88070 |
| fixed | Cycle:Dose | 0.324581683 | 0.78335937 | 0.41434582 | 183.7890 | 0.67910 |
